# Supplementation with a Bioactive Melon Concentrate in Humans and Animals: Prevention of Oxidative Damages and Fatigue in the Context of a Moderate or Eccentric Physical Activity

**DOI:** 10.3390/ijerph17041142

**Published:** 2020-02-11

**Authors:** Marion Saby, Audrey Gauthier, Sandy Barial, Laure Egoumenides, Bernard Jover

**Affiliations:** 1 EA7288 UFR Pharmacie, Université de Montpellier, CEDEX 5, 34093 Montpellier, France; marion.saby@umontpellier.fr (M.S.); sandy.barial@umontpellier.fr (S.B.); 2 Bionov Research, 939 rue de la croix verte, 34090 Montpellier, France; audrey.gauthier@bionov.fr (A.G.); laure.egoumenides@bionov.fr (L.E.); 3 PhyMedExp, INSERM CNRS, Université de Montpellier, IURC, CEDEX 5, 34295 Montpellier, France

**Keywords:** physical activity, oxidative stress, superoxide dismutase, eccentric exercise, melon concentrate, fatigue, PGC-1α, magnesium

## Abstract

Exercise is recognized to provide both physical and psychological health benefits. However, oxidative stress can occur and induce muscular damages. SOD B^®^; M is a melon concentrate, well known to counteract oxidative stress and prevent its side effects. The present study aimed to evaluate the potential of the melon concentrate in the context of both a strong and isolated effort associated with deleterious effects, and a moderate and regular physical activity considered as beneficial. First, a preclinical study was set up on rats to evaluate its potential on the prevention of damages induced by an eccentric exercise. Secondly, the combined effect of the melon concentrate and a regular standardized physical training was studied on the overall physical condition of healthy subjects in a randomized, double-blind, placebo-controlled trial. Repeated measures Analysis of Variance (ANOVA), student’s t test and Mann–Whitney test were used for statistical analyses. Melon concentrate helped to prevent gastrocnemius damages induced by the eccentric exercise. It allowed a reduction of fibrosis by approximately 38% and a reduction of Tumor Necrosis Factor- α (TNF-α) plasma level by 28%. This supplementation also induced a rearrangement of myosin fibers and an increase in PGC-1α plasma level. In the clinical study, melon concentrate was able to decrease oxidative stress and C-Reactive protein (CRP) plasma level. Besides, magnesium (Mg) plasma level was higher in the context of a regular training performed by healthy subjects supplemented with the melon concentrate. Therefore, the melon concentrate allowed a better adaptation to effort linked to PGC-1α activation: a regulator of energy metabolism. The antioxidant properties of the melon concentrate and its ability to mobilize magnesium also suggest that the supplementation could induce a better resistance to fatigue and recovery during regular physical activity.

## 1. Introduction

A regular physical activity has long been recognized as a positive health behavior, known to provide lifelong benefits such as a reduced risk of cardiovascular disease, cancer, and diabetes, but also numerous psychological benefits [1,2]. It is all the more important that modern lifestyle behaviors, which include the highest ever level of sedentarism, are recognized risk factors for chronic disorders, even responsible for a shift in disease patterns [3]. However, whereas physical activity is strongly recommended, it is also well recognized that prolonged and intense exercise is responsible for Reactive Oxygen Species (ROS) overproduction [4,5,6].

ROS are highly reactive molecules but natural by-products of normal cellular metabolism. Whereas a tightly regulated production of ROS is beneficial for normal physiological functions, an excessive production may have several detrimental effects. When an imbalance between their production and detoxification sets in, oxidative stress occurs and with it cells and tissues damages, especially in the muscles and joints [7,8,9]. To assure its oxidative balance, the body constantly acts against the formation of pro-oxidants through different defense mechanisms either enzymatic (superoxide dismutase or SOD, catalase and glutathione peroxidase), or non-enzymatic (vitamins E and C, β-carotene, uric acid and flavonoids, etc.) [6,10]. 

Although regular physical training is normally beneficial for fatigue and recovery, oxidative stress is likely to result in poor adaptation to effort and reduced training effectiveness. Therefore, antioxidant supplementation for athletes seems to be an interesting way to counteract oxidative stress and its side effects.

SOD B^®^; M, a melon concentrate supplement rich in Superoxide Dismutase (SOD), has proven antioxidant and anti-inflammatory effects through a specific mechanism of action [11]. In particular, beneficial effects have been observed on markers of muscular integrity [12], cartilage integrity and inflammation of race horses supplemented with the melon concentrate [13], as well as on running performance and muscle mass of extensor digitorum longus in aged mice [14]. Additionally, melon concentrate has been shown to allow a cardiac protection of the spontaneously hypertensive rats [15,16], as well as several other benefits on diverse oxidative stress associated disorders [17,18,19].

Knowing the involvement of oxidative stress in physical exercise and these previous results, a two-part study was set up in order to further investigate the potential of SOD B^®^; M supplementation on the problematic of oxidative stress in physical activity. In particular, the idea was not only to confirm the relevance of a melon concentrate supplementation in the context of an intense physical activity, but also to determine the degree of health benefits that could be observed on an ordinary population taking a moderate exercise. As a first step, the effect of the melon concentrate was assessed in rats submitted to an eccentric exercise: an unaccustomed and intense exercise known to cause muscle damages, Delayed-Onset Muscular Soreness (DOMS), as well as a high oxidative stress [20,21,22,23]. For that, we followed the protocol described by Armstrong et al. [24] with minor changes. In order to screen the global potential of the supplementation, we then studied the effect of the melon concentrate and a regular standardized physical training, considered beneficial, on the overall physical condition of healthy subjects resuming sport. 

## 2. Materials and Methods 

### 2.1. Preparation and Characterization of the Melon Concentrate

SOD B^®^; M (Bionov, Avignon, France) is a dried melon juice concentrate particularly rich in SOD, which results from a patented process. Briefly, the pulp of a specific proprietary and non-GMO melon variety (Cucumis melo L.) is separated from skin and seeds and crushed before centrifugation. Then the melon juice undergoes filtration and concentration steps. Lastly, the obtained melon juice concentrate is freeze-dried. For oral administration, this dried melon juice concentrate is coated with palm oil in order to preserve its activity from the digestive enzymes secreted above the small intestine. Detailed information about the antioxidant content of this melon concentrate has been previously published [25]. 

### 2.2. Preclinical Study Design

#### 2.2.1. Animals and Experimental Design

The present animal experiment complied with the European and French laws (permit numbers D34-172-25 and 34,179) and conform to the Guide for the Care and Use of Laboratory Animals published by the National Institute of health (National Academies Press US, Eighth edition, 2011).

A rational approach was used in order to reduce the number of animals involved in the study. Considering the statistical analyses planned (analysis of variance preceded by a normality test), n = 9 animals per group was considered appropriate in order to attain sufficient statistical power (alpha risk at 0.05).

Thirty-six Sprague-Dawley (Janvier, le Genest-St-Isle, France) of 9 weeks-old were used in the present experiments. They were housed at 22 ± 1 °C, subjected to a 12 h light/12 h dark cycle with free access to both food (A04, SAFE, Augy, France) and tap water. After one week of an adaptation period, rats were subjected to 3 days of familiarization with treadmill. During this familiarization phase, the animals ran on the treadmill for 5 min at 9 m/min the first day then 10 min at a speed of 12 m/min the two following days (non-inclined treadmill, which corresponds to an isometric work). On the fourth day, the maximum aerobic running speed (VMA) was determined. The exercise was performed on a non-inclined treadmill and consisted of an initial period called “warm up” of 5 min at 15 m/min, followed by an increment phase during which the speed was increased by 2 m/min every minute. The VMA was determined when the animal was not able to maintain the running speed. Then, rats were randomly divided into four groups (n = 9 in each) and subjected or not to an eccentric exercise with or without melon supplementation. In previous studies, melon concentrate supplement demonstrated antioxidant and anti-inflammatory effects on diverse pathologic models for daily doses between 4 U SOD [15,16] and 16 U SOD (rat equivalent dose) [17,18]. Besides, a study conducted on race horses submitted to intense training sessions [13] demonstrated the efficacy of the ingredient at a daily intake of 16 U SOD (rat equivalent dose). Therefore, the melon concentrate was given at the dose of 16 U SOD, once a day during 5 days as a pellet mixed with food. 

The first group received no treatment and was not exercised (Control group: C). The second group received no treatment and was subjected to an eccentric exercise (Eccentric Exercise group: EE). The third group received the melon concentrate for 5 days with no exercise (Control treated group: CT) and the fourth group received the melon concentrate for 5 days and was subjected to an eccentric exercise (Eccentric Exercise + treatment group: EET). The eccentric exercise was performed on a rodent treadmill and the four groups completed a 3 days familiarization period with the treadmill and one session of VMA test before the start of the supplementation. Then, C and CT groups were not subjected to exercise anymore. After the last supplementation, only exercised rats without or with treatment (EE and EET group) were immediately subjected to an eccentric exercise using the protocol described by Armstrong et al. [24,26] with minor changes: rats were subjected to intermittent downhill using treadmill with a —16° inclination at the speed of 16 m/min for a total of 90 min. Therefore, the protocol included eighteen 5-min bouts separated by 2-min rest period. 

At the end of the experimental period, rats were weighed and anesthetized (ketamine and xylazine, 75 and 25 mg/kg). Then, 4 mL of blood was sampled in all rats by cardiac puncture, centrifuged (2000× *g*, 10 min, 4 °C), and plasma was stored at −80 °C until analysis. Gastrocnemius muscle was excised, sectioned and stored at −80 °C or fixed in formalin 10% until histological analysis.

#### 2.2.2. Histology of the Gastrocnemius Muscle

Paraffin-embedded gastrocnemius muscle was cut in 5 µm slices sections and mounted on Superfrost Plus glass slides (Menzel, Braunschweig, Germany).

For morphological analysis, slices were stained with hematoxylin-eosin. The number of muscle fibers was evaluated and the myocyte size was determined by measuring the shortest transverse diameter. All the analyses were performed in a blind fashion by three different observers on at least 5 transverse sections per muscle using image analysis software (ImageJ, Bethesda, MD, USA). 

For fibrosis determination, gastrocnemius sections were stained with 0.1% picrosirius red and mounted in Eukitt medium. Fibrosis was quantified in five to ten given fields per animal, and expressed as the percentage of fibrous tissue area stained with picrosirius red. 

#### 2.2.3. Gastrocnemius Western Blot Analysis

The muscle protein extraction was carried out on ice in 20 mM of Tris buffer (pH 6.8) containing 150 mM sodium chloride, 1 mM Ethylenediaminetetraacetic acid (EDTA), 1% Triton 20%, 0.1% sodium dodecyl sulfate (SDS), 1% protease inhibitor cocktail (Sigma-Aldrich, Darmstadt, Germany). After centrifugation (5500× *g* 15 min, 4 °C), the supernatant was collected and the extracted tissue proteins were then separated by SDS polyacrylamide gel electrophoresis. Equal amounts of proteins were loaded onto a 5% or 15% acrylamide gel with a 4% stacking acrylamide gel. Migration was conducted in a Tris-glycine-SDS buffer. After separation, proteins were transferred onto nitrocellulose membranes (Whatman, Germany). 

Myosin proteins were detected by Western blot analysis. The following primary antibodies against rat skeletal slow myosin heavy chain (Sigma-Aldrich, Darmstadt, Germany), skeletal fast myosin heavy chain (Sigma-Aldrich, Darmstadt, Germany), and the control protein tubulin (R&D Systems, Minneapolis, MN, USA) were used. Expression of tubulin was used for checking the equal protein load across gel tracks. Secondary antibodies (Sigma-Aldrich, Darmstadt, Germany), coupled with alkaline phosphatase, were used for revealing the primary antibodies. Western blotting was performed according to Amersham ECL select protocol (GE Healthcare, Velizy-Villacoublay, France) and was acquired with a chemiluminescence detection system (Chemi-smart 5000, Vilbert Lourmat, Marne-la-Vallée, France). Image analysis (ImageQuant TL, GE Healthcare, France) was used for quantification after standardization within membranes by expressing the density of each band of interest relative to that of tubulin in the same lane. Results are then expressed as percent of values obtained in untreated animals.

#### 2.2.4. Plasma Immunoassay Measurements

Plasma PGC-1α and TNF-α levels were assessed using enzyme immunoassay kits from Mybiosource (San Diego, CA, USA) and R&D Systems (Minneapolis, MN, USA). The PGC-1α immunoassay kit used gastrocnemius nuclear extract and absorbance was measured at 450 nm using a microplate reader. TNF-α immunoassay kit used gastrocnemius extract and absorbance was measured using the absorbance difference 450 nm–540 nm using a microplate reader. Results are expressed as picograms of PGC-1α or TNF-α per milligrams of total proteins.

### 2.3. Clinical Study

#### 2.3.1. Methods: Tools for the Physical Evaluation

The investigation focused on the effects of the melon concentrate on physical condition improvement of healthy subjects undergoing a physical training program. The main criterion of this study concerned the improvement of the physical condition whereas secondary criteria assessed changes in physical performance, quality of life and tiredness, inflammation, ionic modifications and changes in blood oxidative status.

The overall physical condition improvement was evaluated with the Ruffier test [27,28]. In this validated test, subjects completed 30 flexions in 45 s. Three measurements of Heart Rate (HR) were taken: pre-test resting HR, HR immediately after performing the flexions, and recovery HR 60 s post-test. Those three HRs were then used to calculate the Ruffier index. 

The modification of physical performance was assessed using the half Cooper test [29], an exercise consisting of running the biggest distance possible (D) in 6 min. The half Cooper allows us to evaluate the Maximal Aerobic Speed (MAS), described as the smallest running speed from which a person uses the maximum of O2 or reaches the VO_2max_ (maximum volume of oxygen that the body can use during an effort). MAS is calculated with the formula MAS = D/100 and VO_2max_ as follows: VO_2max_ = MAS × 3.5 mL·min^−1^·kg^−1^ [30].

Besides, the Resting Heart Rate and the Maximum Heart Rate (MHR) were measured at rest and during the half Cooper test respectively. 

The influence of a physical activity on the quality of life and fatigue was evaluated with 2 auto evaluation surveys: SF 36^®^; and Prevost subjective fatigue scale. SF 36^®^; (The Health Institute, Boston, MA, USA) allows the measurement of eight aspects of the quality of life: general physical and mental health state, physical and social functioning, physical and emotional health, pain, and vitality [31]. The SF 36^®^; questionnaire is composed of 36 questions with a total score from 36 to 149 and allow us to obtain 8 scores in every aspect. 

Prevost subjective fatigue scale is a questionnaire intended to measure the impact of stress on physical fatigue of the subjects [32]. The subject has to score from 1 to 7 the following impact points on their fatigue: global fatigue perceived level, muscle pain, sleep troubles and stress.

#### 2.3.2. Study Design

The clinical trial was an intervention study based on the individual evaluation scales described above and below. It was a monocentric study performed from November, 2016 to March, 2018. The protocol followed was a controlled clinical study vs. placebo, randomized, and double blind during 56 days ± 8 days. It was approved by the Comité de Protection des Personnes (CPP) Sud-Ouest et Outre-Mer 1 on the 29th of August 2016 and the Agence National de Sécurité du Medicament (ANSM) on the 26th of April 2016 (clinical trial registration: NCT02880657). It was also declared to the Commission Nationale de l’Informatique et des Libertés (CNIL). The calculus of the sample size needed for the clinical study was done considering that the combination of training and SOD B^®^; M would decrease the Ruffier by 1 point with a variability of ± 1 compared to training alone [33]. In this way it was assessed that 21 subjects per group would be needed to have an adequate statistical power for 90% potency and an alpha risk equal to 0.05. Taking into account dropouts estimated at 17%, it was planned to randomize a total of 50 subjects. A call for volunteers was made in the clinical investigation centers and the volunteers for the study were pre-screened by the investigators. 

The inclusion criteria were to be a man between 30 and 55 years old, to have a Body Mass Index between 18.5 and 29.5 kg/m^2^, to have an insufficient/average adaptation to effort corresponding to a Ruffier index between 8 and 12 (limits included), to have a stable weight (variation < 5% over the last 3 months), a stable diet over the past 3 months, normal biological exams, a blood pressure lower than 140/90 mm Hg and no contraindication to the practice of running. 

Subjects with a familial dyslipidemia or treated with statins, and subjects presenting pathologies likely to distort the results of the study or to interfere with the specific assays (arterial hypertension, type II diabetes, chronic respiratory pathology(s), rheumatic or orthopedic diseases) were excluded. Treatment with drugs that may have a doping effect (glucocorticoids, narcotics, stimulants, etc.) or consumption of psychoactive substances (cannabis, heroin, cocaine, ectasia, amphetamines) were also part of exclusion criteria.

After a pre-selection phone interview, a pre-inclusion visit (V1) took place. This visit included, as a first step, the signing of the consent and the verification of the clinical inclusion/exclusion criteria (including the Ruffier), at the Clinical Investigation Center (CIC).

Directly after V1, subjects conducted a baseline test session including the Prevost test, the SF 36^®^; survey and the half Cooper test.

The second visit (V2) corresponded to the inclusion visit. Various blood parameters were determined after a test training session and used as baseline values. Forty-one volunteers were definitively included and participated in the clinical trial. The volunteers were assigned by randomization into two groups of 21 subjects for the treated group and 20 subjects for the placebo group (P) and given a food supplement or a placebo for 56 days ± 8 days. The capsules were indistinguishable and were administered in a double-blind approach. Volunteers were given two small hard capsule per day corresponding to 40mg or 560 U SOD and excipients for the active supplement, and excipients only for the placebo. This dose was chosen in correlation with the preclinical study. 

The training phase then started: this phase was composed of 16 standardized training sessions, performed over a period of 8 weeks, twice a week, with 2–3 resting days between each session. Those training sessions aimed to improve the physical condition of the subjects. Training session took place on a treadmill. Endurance and aerobic sessions of variable duration were alternated during this physical training. All trainings were supervised by the same referent trainer in order to harmonize training follow-up. Before each session, the coach checked the ability of the subject to perform the training (injury, tiredness, etc.) and collected the potential adverse events. 

The blood samples were used for complete blood count. Biochemical analyses were also performed to evaluate the ionic changes. C-Reactive Protein (CRP) level was assessed as a marker of inflammation and the overall antioxidant defense potential was estimated via the KRL (Kit Radicaux Libres) test (“SPIRAL” laboratories, Couternon, France) which is a biological test of blood resistance based on free radical induced haemolysis. The latter test measures the global antioxidant capacity (enzymatic and non-enzymatic) of erythrocytes against a standardized assault with free radicals. Therefore, it allows the dynamic evaluation of the overall antioxidant defense potential of an individual. All the blood analyses and tests were realized at the Institut Fédératif de Biologie - CHU Toulouse Purpan.

A follow-up visit (V3) was conducted midway, after the 8th training session at the CIC. V3 aimed to check treatment adherence, potential adverse effects, and physical condition improvement at half training with the Ruffier test.

**Figure 1:**
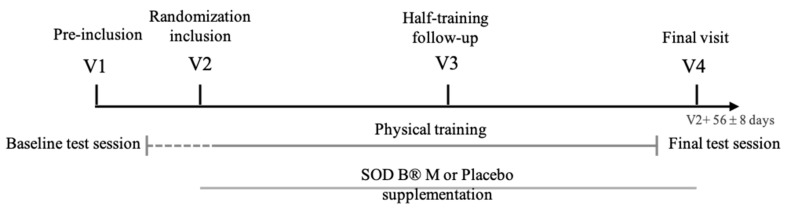
Schematic representation of the clinical study design. V1 includes the Ruffier test, Baseline test session includes the Prevost test, SF 36^®^; survey and Half Cooper test. V2 comprises a blood sampling, V3 includes the Ruffier test, Final test session includes the Prevost test, SF 36^®^; survey and Half Cooper test. V4 includes the Ruffier test and blood sampling.

The 16th and last training session was directly followed by a final test session identical to the baseline one, in order to evaluate the evolution of the Prevost and SF 36^®^; scores as well as parameters of the half Cooper test. Results were attached to the final visit (V4) which took place 2 days later. At V4, subjects conducted the Ruffier test, blood was sampled and a clinical exam was performed. The clinical study design is schematized on Figure 1.

### 2.4. Statistical Analyses

Values from preclinical study are presented as means ± SEM. Statistical analysis of the data was carried out using GraphPad Prism software (La Jolla, CA, USA) by one-way ANOVA followed by Mann–Whitney’s test. P-values less than 0.05 were considered to be significant.

The clinical data were expressed as means ± SEM. The comparison between supplement and placebo was carried out on the differences V3–V1, V4–V1, V4–V3 and V4–V2 using either student’s t test for intragroup analysis and an Analysis of Covariance (ANCOVA), student’s t test or Mann–Whitney test for intergroup analysis. The software used for those analyses was SAS^^®^;^ version 9.4 (SAS, Cary, NC, USA). *p*-values less than 0.05 were considered to be significant.

## 3. Results

### 3.1. Preclinical Study

#### 3.1.1. Melon Concentrate Supplementation Reduced Myocytes Damages Induced by Eccentric Exercise

**Figure 2:**
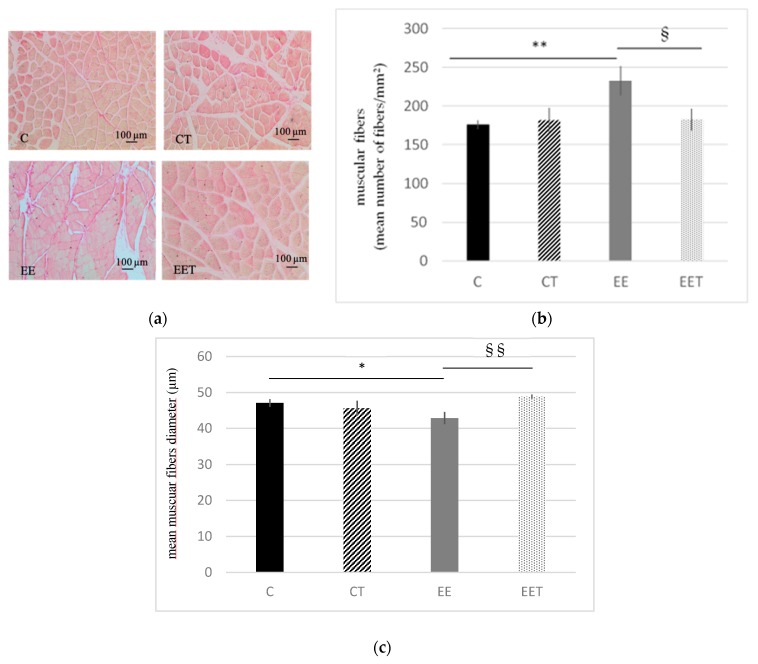
Influence of melon concentrate supplementation on the number and diameter of muscular fibers (gastrocnemius muscle). (**a**) Hematoxylin-Eosin Staining (HES) histochemical staining of muscular fibers. (**b**) muscular fibers were quantified on five transverse sections per muscle. Results are expressed in number of fibers/mm2 of muscle ± SEM. ** *p* < 0.01 effect of the eccentric exercise compared with control group, § < 0.05 effect of melon concentrate supplement compared with the EE group. (**c**) muscular fibers diameter was measured on five transverse sections per muscle ± SEM. Results are expressed in µm. * *p* < 0.05 effect of the eccentric exercise compared with control group, §§ < 0.01 effect of melon concentrate supplement compared with the EE group.

As shown in Figure 2, eccentric exercise induced a 32% approximate increase in the number of muscular fibers (C 176 ± 5.5 vs. EE 233 ± 18 fibers/mm^2^, *p* < 0.01) and a 9% decrease of their diameter (C 47 ± 1 vs. EE 43 ± 2 µm, *p* < 0.05). There is no significative difference between C and CT groups. On the other side, with no significant difference between C and EET group, melon concentrate supplementation was able to fully preserve muscular fibers from damages induced by eccentric effort. 

#### 3.1.2. Melon Concentrate Supplementation Reduced Muscular Fibrosis Induced by Eccentric Exercise

**Figure 3:**
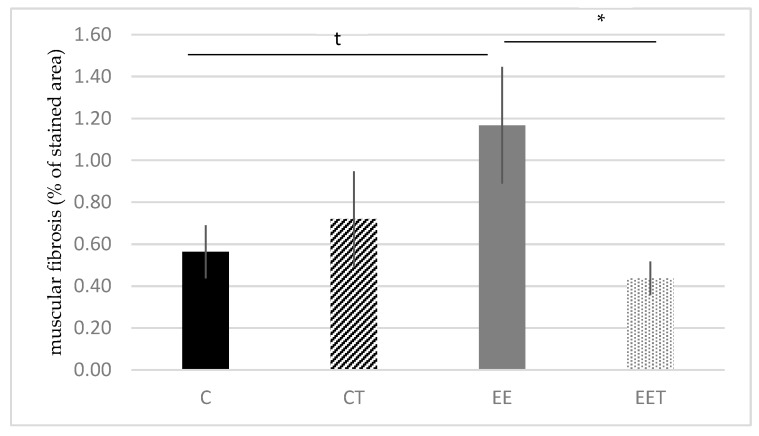
Influence of melon concentrate supplementation on the muscular fibrosis (gastrocnemius muscle). Muscular fibrosis measured on five transverse sections per muscle. Results are expressed as percentage of fibrous tissue area stained with picrosirius red ± SEM. t *p* < 0.1 effect of the eccentric exercise compared with control group, * < 0.05 effect of melon concentrate supplement compared with the EE group.

The evaluation of muscular collagens is shown in Figure 3. The staining tends to increase in EE, compared to C group (EE 1.17 ± 0.28 vs. C 0.56 ± 0.13% of stained area, *p* < 0.1). Melon concentrate supplementation allowed us to significantly reduce this alteration by 38% (*p* < 0.05) and to preserve muscular fibers from fibrosis, in the context of an eccentric exercise. No difference was observed between C and CT groups.

#### 3.1.3. Melon Concentrate Supplementation Induced a Rearrangement of Myosin Fibers

**Figure 4:**
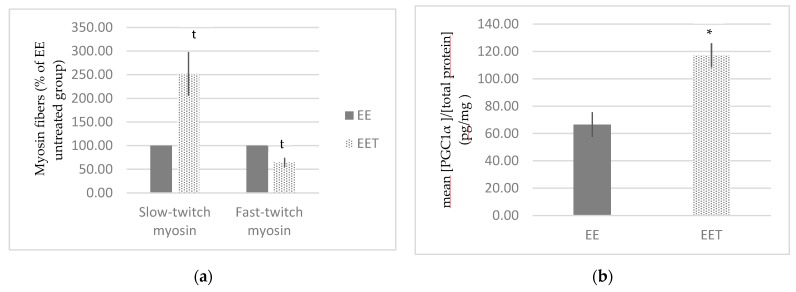
Influence of melon concentrate supplementation on the gastrocnemius protein expression of myosin fibers and the PGC1α plasma level. (**a**) Quantification was made after standardization within membranes by expressing the density of the band of slow-twitch or fast-twitch myosin relative to that of tubulin in the same lane. Results are then expressed as relative change from untreated EE band intensity ± SEM. t *p* < 0.1 effect of melon concentrate supplement compared with the EE group. (**b**) Results are expressed as mean [PGC1α]/[total protein] (pg/mg) ± SEM. * *p* < 0.05 effect of the melon concentrate supplementation in the context of an eccentric exercise compared with EE group.

Figure 4 represents the rearrangement of myosin fibers following melon concentrate supplementation. There was a trend that fast-twitch myosin fibers were reduced by around 38% and slow-twitch myosin fibers were 2.5 times increased in EET group in comparison with EE group (*p* < 0.1). Melon concentrate supplementation thus induced a rearrangement of myosin fibers in the context of an eccentric exercise. No difference was observed without eccentric exercise.

#### 3.1.4. Melon Concentrate Supplementation Induced an Increase in PGC-1α Plasma Level

There was no difference in PGC-1α plasma level between group C and group EE (69.07 ± 6.29 vs. 66.50 ± 9.07 pg/mg of proteins). On the other side, as displayed in Figure 4, we observed a significant increase by approximately 76% in PGC-1α plasma level following melon concentrate supplementation, in the context of eccentric exercise (EE 66.50 ± 9.07 vs. EET 117.14 ± 8.80 pg/mg of proteins, *p* < 0.05).

#### 3.1.5. Melon Concentrate Supplementation Induced a Decrease of TNF-α Plasma Level

**Figure 5:**
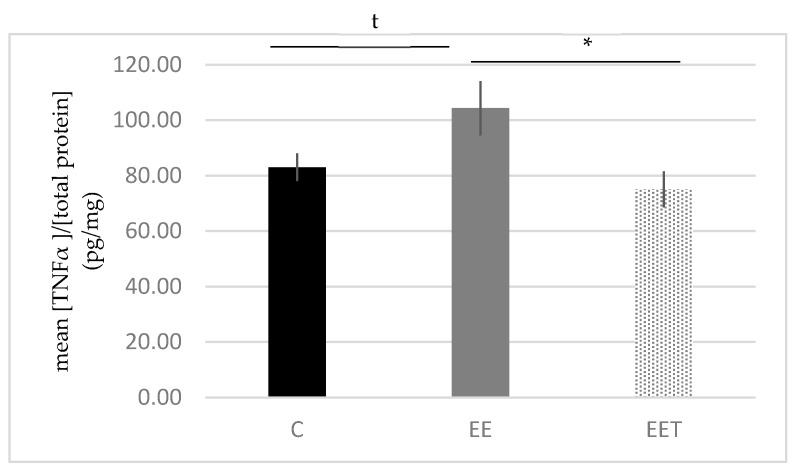
Influence of melon concentrate supplementation on the TNFα plasma level. Results are expressed as mean [TNFα]/[total protein] (pg/mg) ± SEM. t *p* < 0.1 effect of the Eccentric exercise compared with Control group. * *p* < 0.05 effect of the melon concentrate supplementation compared with EE group.

Figure 5 presents the evolution of TNF-α plasma level. Eccentric activity tends to increase TNF-α level by 26% in the plasma (EE 104.32 ± 9.77 vs. C 83.03 ± 4.97 pg/mg of proteins). In addition, melon concentrate supplementation was able to significantly lower by 28% this biomarker (EET 75.04 ± 6.51 vs. EE 104.32 ± 9.77 pg/mg of proteins, *p* < 0.05). The melon concentrate supplementation allowed to maintain a normal TNF-α plasma level in the context of an eccentric effort (C vs. EET).

### 3.2. Clinical Study

#### 3.2.1. Study Population

Forty-one volunteers aged between 30 and 53 years old (mean 38.8 years old) were recruited and randomized into two test groups (n = 21 for melon concentrate supplemented group (verum) and n = 20 for placebo group). Three subjects were excluded during the course of the study and therefore 38 subjects were analyzed. There were no statistical differences between the two groups at baseline.

#### 3.2.2. Physical Condition Improvement

As shown in Table 1, there was a highly significant decrease of the Ruffier index between V1 and V3 (*p* < 0.001), observed both in the treated and in the placebo group, following intragroup analysis. The score kept decreasing during the second part of the study (V3 vs. V4) but in a non-significant manner. Overall (V1 vs. V4), both groups experienced a significant intragroup decrease of the index but with no intergroup difference. 

#### 3.2.3. Physical Performance Improvement

As presented in Table 1, HR and MHR decreased in a non-significant manner between V1 and V4 whatever the group. Besides, MAS and VO_2max_ were increased between V1 and V4. Even if there is no significant difference in MAS and VO2_max_ evolution with intergroup analysis, those parameters were significantly increased in the placebo group and the supplemented group following intragroup comparison (*p* < 0.001). 

#### 3.2.4. Quality of Life: Prevost Subjective Fatigue Scale and SF 36^®^; Health Survey

Regarding the Prevost scale, the mean of total index at baseline was 11. As reported on Table 1, Prevost score did not change in any groups between V1 and V4 using intergroup statistical analysis. However, intragroup analysis revealed a downward trend of the score in the treated group (*p* = 0.068). There was no significant difference between the two groups using intergroup analysis. However, intragroup analysis shown that physical and mental scores (SF 36^®^;) were both increased in the supplemented group (respectively *p* = 0.001 and *p* = 0.006) whereas only the mental score was improved in the placebo group (*p* = 0.001).

#### 3.2.5. Melon Concentrate Supplementation Decreased Oxidative Stress

**Figure 6:**
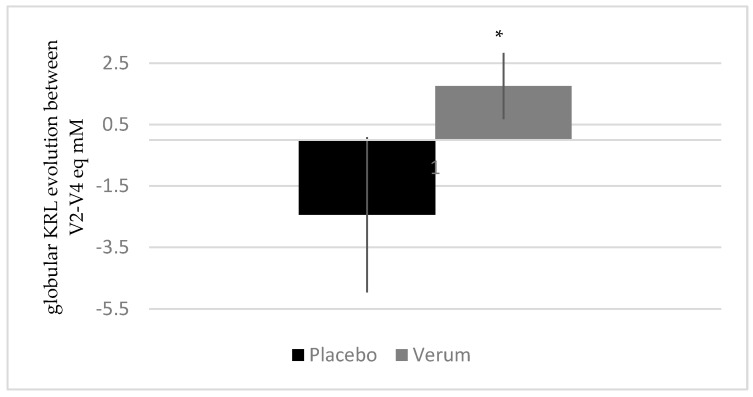
Influence of melon concentrate supplementation on the oxidative defenses. * *p* < 0.05 effect of the melon concentrate supplementation compared with placebo group.

Figure 6 shows the evolution of globular KRL between V2 and V4 for both groups. As presented, the erythrocyte resistance to hemolysis increased in the treated group (1.75 ± 4.04 eq mM) whereas it decreased in the placebo group (−2.44 ± 8.74 eq mM), with no intragroup variation but a significant intergroup difference (*p* < 0.05). 

#### 3.2.6. Melon Concentrate Supplementation Tended to Decrease CRP Plasma Level

**Figure 7:**
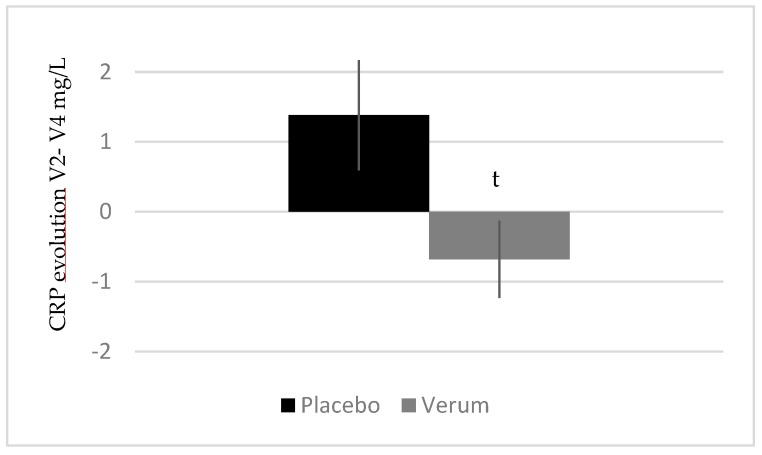
Influence of melon concentrate supplementation on the CRP plasma level. Results are expressed as [CRP] (mg/L) ± SEM. t *p* < 0.1 effect of the melon concentrate supplementation compared with placebo group.

Figure 7 presents the evolution of CRP plasma level between V2 and V4. Results show that CRP slightly increased in the placebo group (1.38 ± 3.35 mg/L) and slightly decreased in the treated group (−0.68 ± 2.34 mg/L) (non-significant intragroup analysis). These opposed progressions of the two groups allow us to observe a trend in intergroup analysis (*p* < 0.1). 

#### 3.2.7. Melon Concentrate Supplementation Modified the Platelet Level and Induced an Increase in Magnesium Plasma Level 

**Figure 8:**
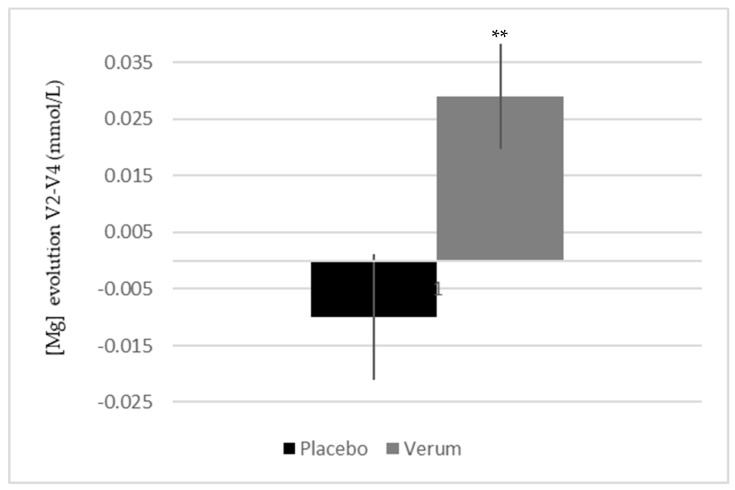
Influence of melon concentrate supplementation on the magnesium plasma concentration. Results are expressed as [Mg] (mmol/L) ± SEM. ** *p* < 0.01 effect of the melon concentrate supplementation compared with placebo group.

There was a significant decrease of the platelet level between V2 and V4 in the placebo group (−5.8 ± 29.3 G/L) in comparison to the treated group where the platelet level remained stable (1.2 ± 20.2 G/L) (*p* < 0.05). There were no changes regarding the evolution of the other blood cells. Regarding blood ionic parameters, only magnesium (Mg) plasma level was impacted by the supplementation, with a significant intragroup and intergroup increase in the treated group between V2 and V4 compared to placebo (verum 0.029 ± 0.038 vs. placebo −0.010 ± 0.047 mmol/L; *p* < 0.01 in both cases) (Figure 8). 

## 4. Discussion

The present preclinical study aimed to evaluate the potential of the melon concentrate on the prevention of damages induced by an eccentric exercise in rats. It is demonstrated that strong and unaccustomed eccentric exercise is associated with muscle damage and DOMS [23,24,34,35,36,37,38,39]. Eccentric contractions are characterized by the lengthening of the muscle-tendon complex; since fibers do not change in volume when they deform, they reduce in diameter as they increase in length [20,21,22,23]. In this preclinical study, the eccentric exercise was, as it could be expected, associated with enhanced muscular fibrosis, an increase in the density of muscular fibers suggesting an increase in their number and a reduction in their diameters in the gastrocnemius, as well as an increase in TNF-α plasma level. Conversely, the data demonstrated that the supplementation enabled all four parameters to be fully normalized. These results confirmed and extended previous reports on the beneficial effects of melon concentrate supplementation in models subjected to stressful conditions, including humans with perceived stress, horses subjected to an intense physical exercise, obese hamsters or spontaneously hypertensive rats [13,15,17,19,40].

It was evidenced in a previous unpublished transcriptomic study that the expression of PGC-1α gene was significantly increased following a melon concentrate supplementation. Therefore, special attention was paid to this key regulator of energy metabolism.

The present study confirmed that melon concentrate was able to induce an increase in PGC-1α plasma level. PGC-1α is well known to stimulate mitochondrial biogenesis, but also to promote the remodeling of muscle tissue by modulating fiber-type composition [41,42,43]. Interestingly, the study evidenced that the supplementation tended to induce a decrease in fast-twitch myosin fibers and an increase in slow-twitch myosin fibers following the eccentric exercise. Physiologically, fast-twitch fibers use glycolytic metabolism allowing fast and powerful contractions during a short period of time. On the other hand, slow-twitch myosin fibers are rich in mitochondrial network and with good vascularization, they allow slower and less powerful contraction, but more energetically efficient [44,45,46]. It is therefore possible to hypothesize that melon concentrate, through PGC-1α transcriptional coactivator, promoted the transition from fast-twitch to slow-twitch fibers in order to encourage a more energetically efficient metabolism. This would allow an improvement of endurance, performance, adaptation to effort as well as resistance to fatigue. 

Besides, PGC-1α has also been involved in the antioxidant defense system and several studies indicate that it is required for the induction of many ROS-detoxifying enzymes [47,48,49,50,51]. At this point it is interesting to note that according to previous studies, the melon concentrate provides its antioxidative capacity through a stimulation of endogenous antioxidant defenses [16,25,52]. PGC-1α could be a link in the cascade of reaction responsible for this induction, and add new perspectives in the understanding of the melon concentrate mechanism of action. 

Overall, the preclinical data demonstrated the efficacy of the melon concentrate in the context of an eccentric exercise, responsible for muscular damages. Simultaneously, the findings on PGC-1α and myosin fibers brought us a different perspective. In particular, it suggested that melon concentrate not only prevents damages induced by an eccentric effort, but also benefits the adaptation to effort in the context of a moderate and regular physical activity. The aim of the clinical study was here to evaluate the effect of the melon concentrate on the adaptation to effort and physical performance and to assess the overall potential of the supplementation in two types of exercises. For this, a healthy volunteer population, with low or medium capacity of recovery and starting a moderate physical activity, was the most relevant. Fifty healthy subjects were planned for this clinical trial but only 41 subjects reaching inclusion criteria were recruited and 38 subjects were analyzed. Even if initial population was not reached, statistical analyses were still compliant.

First of all, the training followed during this study resulted in a clear improvement of physical parameters (MAS and Ruffier index), similarly observed in the two groups. More specifically, a fast and significant reduction of the Ruffier index was observed between V1 and V3, whereas only a slight further improvement occurred during the second half of the trial (between V3 and V4). These results indicated that training had a great and rapid beneficial impact on subjects’ physical condition but also suggest that intermediary assessments after a shorter training period (between V1 and V3) would have increased the probability to observe an effect of the supplement.

Among the biomarkers assessed, the antioxidant capacity of the ingredient was analyzed through the KRL, a validated and widely used test assessing blood resistance to a free radical attack [53]. Whereas no effect of the training was observed, the supplementation significantly increased the anti-free radical protection of erythrocytes compared to placebo. This indicated a better resistance to oxidative stress in the context of a moderate-intensity physical training. Similar results were previously obtained in Standardbred trotters subjected to intense and repeated training sessions after 60 days of supplementation [12]. These results supported the antioxidant effect of the melon concentrate previously demonstrated [16,17,18] and suggest that this prevention of oxidative damages should be applicable in both cases of an intense and a moderate activity.

The strong relationship between physical activity and inflammation has often been reported in the literature. It is well established that physical activity can increase inflammatory markers or conversely decrease them, depending on the type, the intensity and the duration of the exercise [54]. Here, training was regular and of moderate intensity, which explains that no significant increase in CRP level was observed in placebo group. On the other side, a downward trend was observed in the supplemented group, thus indicating a reduction of systemic inflammation. This finding is consistent with that of Barbé et al. [13], who observed a reduction of plasma prostaglandin E2 level in athletic horses after melon concentrate supplementation. In this particular case, the supplementation enabled to lessen the initial inflammatory state and to maintain this inhibition during and after the training session, whereas the control group experienced a significant increase in inflammation. These data confirmed the anti-inflammatory potential of the ingredient, no matter the type of exercise and the level of inflammation induced [13,18].

Another important finding was the effect of supplementation on the plasmatic Mg level. Mg is an essential mineral involved in many enzymatic systems, energetic metabolism, protein biosynthesis, as well as neuro-muscular excitability [55,56,57,58]. Several reports have shown that prolonged strenuous exercise is accompanied by hypomagnesemia. Indeed, the body responds to exercise by redistributing Mg to locations with increased metabolic need, for processes such as energy production or counteracting oxidative stress [55,56,59]. A decrease in Mg level is then characterized by a general fatigue and a tense state, both muscularly and nervously [55,56,57,58]. 

In this study, the concentration of plasmatic Mg was significantly increased in supplemented group, whereas it remained stable in the placebo one. This result may be explained by the antioxidant capacity of the melon concentrate: by decreasing oxidative stress, the melon juice concentrate limits the enhanced need for Mg of the most metabolically active cells, as well as its redistribution. This protective effect on Mg level could be correlated with an increased resistance to fatigue and a better recovery, with the implication for example of a delayed lactate accumulation in the muscle [60]. Moreover, these data could explain the positive effects observed on well-being and fatigue.

To that extent, Prevost and SF 36^®^; scales were used, in order to assess a general evaluation of physical and mental condition in subjects. First, the results showed that supplementation tended to reduce Prevost score, thus traducing improved fatigue, muscular pain, sleep troubles and stress levels. Besides, melon concentrate also enabled to increase SF 36^®^; score, with a significant rise observed for both physical and mental sections whereas placebo only had a positive effect on mental score. Those results indicated that the regular training by itself induced a positive effect on mental well-being whereas the supplementation was able to act on the physical sphere of wellness. These beneficial effects of the melon juice concentrate are in line with those reported by Milesi et al. [19] and also by Carillon et al. [61]. In those studies, conducted on subjects with perceived stress and tiredness, even more favorable outcomes were observed, not only on physical well-being but also on stress and mental health. This could suggest that, as already observed in the literature [15,62], a lower baseline is associated with an increased efficacy. Therefore, the supplementation could also be relevant in a population practicing intensive activities or in athletes submitted to both physical and mental tension.

Overall, the combined study of those experiments performing two specific types of exercises, allowing us to collect a large range of complementary data. This original approach enabled to highlight the positive effect of the melon concentrate on several biomarkers, as well as its potential on different types of physical activity. With the benefit of hindsight, the main limitation of this study was that combining the supplementation with a protocol aiming to have beneficial effects made it even more difficult to observe a positive effect of the ingredient. Adding intermediary assessments after a shorter training period could be a means of enhancing this point, and should be considered in a future trial.

## 5. Conclusions

Preclinical study confirmed the positive effect of melon concentrate in presence of stress factors, here an eccentric exercise. In particular, the supplementation helped to prevent gastrocnemius damages induced by the exercise, most probably through a regulation of oxidative stress. The identification of the rearrangement of myosin fibers mediated by PGC-1α oriented the study on the field of adaptation to effort, endurance, performance. The clinical trial that followed demonstrated that melon concentrate also had direct benefits on oxidative stress, inflammation reduction and Mg level protection correlated with fatigue reduction. Altogether, the present results confirm the positive effect of the melon concentrate in two types of exercise: a strong and isolated eccentric effort considered as an unfavorable exercise condition and a moderate and regular physical activity considered as favorable. It also suggests a beneficial effect on the recovery stage following an effort, which deserves further investigation.

## Figures and Tables

**Table 1:** Influence of the melon concentrate supplementation on the evolution of physical performance parameters, physical condition and SF 36^®^; and Prevost scores. Intragroup comparison of Verum and Placebo groups. *** *p* < 0.001, t *p*< 0.1 effect of melon concentrate supplement between V1 and V4 or V1 and V3.

		Placebo	Verum
Evolution V1–V4	HR (b/min)	−3.3 ± 8.1	−2.9 ± 12.2
	MHR (b/min)	−4.33 ± 10.09	−2.35 ± 20.40
	MAS (km/h)	1.75 ± 1.14 ***	1.66 ± 1.24 ***
	VO_2max_	6.14 ± 3.98 ***	5.80 ± 4.34 ***
	Prevost score	−0.8 ± 3.8	−1.4 ± 3.2 ^t^
	SF 36^®^;-Physical score	1.97 ± 3.69	4.8 ± 1.68 ***
	SF 36^®^;-Mental Score	13.04 ± 3.16 ***	8.4 ± 3.14 **
Evolution V1–V3	Ruffier index	−3.00 ± 2.42 ***	−2.78 ± 2.64 ***
Evolution V3–V4	Ruffier index	−0.87 ± 2.42	−1.1 ± 3.05
Evolution V1–V4	Ruffier index	−3.87 ± 2.32 ***	−3.93 ± 2.63 ***

## References

[1] Fentem P.H. (1994). ABC of sports medicine. Benefits of exercise in health and disease. BMJ.

[2] Warburton D.E., Nicol C.W., Bredin S.S. (2006). Health benefits of physical activity: The evidence. CMAJ Can. Med. Assoc. J..

[3] Schulenkorf N., Siefken K. (2019). Managing sport-for-development and healthy lifestyles: The sport-for-health model. Sport Manag. Rev..

[4] Kawamura T., Muraoka I. (2018). Exercise-Induced Oxidative Stress and the Effects of Antioxidant Intake from a Physiological Viewpoint. Antioxidants.

[5] Simioni C., Zauli G., Martelli A.M., Vitale M., Sacchetti G., Gonelli A., Neri L.M. (2018). Oxidative stress: Role of physical exercise and antioxidant nutraceuticals in adulthood and aging. Oncotarget.

[6] Reid M.B. (2001). Invited Review: Redox modulation of skeletal muscle contraction: What we know and what we don’t. J. Appl. Physiol..

[7] Betteridge D.J. (2000). What is oxidative stress?. Metab. Clin. Exp..

[8] Sies H. (1991). Oxidative stress: From basic research to clinical application. Am. J. Med..

[9] Sies H. (1997). Oxidative stress: Oxidants and antioxidants. Exp. Physiol..

[10] Birben E., Sahiner U.M., Sackesen C., Erzurum S., Kalayci O. (2012). Oxidative stress and antioxidant defense. World Allergy Organ. J..

[11] Carillon J., Rouanet J.M., Cristol J.P., Brion R. (2013). Superoxide dismutase administration, a potential therapy against oxidative stress related diseases: Several routes of supplementation and proposal of an original mechanism of action. Pharm. Res..

[12] Notin C., Vallon L., Desbordes F., Leleu C. (2010). Oral supplementation with superoxide dismutase in Standardbred trotters in training: A double-blind placebo-controlled study. Equine Vet. J. Suppl..

[13] Barbé F., Sacy A., Le Treut Y., Lennen C., Lindinger M. (2017). Maintien de l’intégrité musculaire et articulaire: Les bénéfices de la Superoxyde Dismutase végétale.

[14] Chabi B., Pauly M., Carillon J., Carnac G., Favier F.B., Fouret G., Bonafos B., Vanterpool F., Vernus B., Coudray C. (2016). Protective effect of myostatin gene deletion on aging-related muscle metabolic decline. Exp. Gerontol..

[15] Carillon J., Gauthier A., Barial S., Tournier M., Gayrard N., Lajoix A.D., Jover B. (2016). Relaxin and atrial natriuretic peptide pathways participate in the anti-fibrotic effect of a melon concentrate in spontaneously hypertensive rats. Food Nutr. Res..

[16] Carillon J., Rugale C., Rouanet J.M., Cristol J.P., Lacan D., Jover B. (2014). Endogenous antioxidant defense induction by melon superoxide dismutase reduces cardiac hypertrophy in spontaneously hypertensive rats. Int. J. Food Sci. Nutr..

[17] Carillon J., Knabe L., Montalban A., Stevant M., Keophiphath M., Lacan D., Cristol J.P., Rouanet J.M. (2014). Curative diet supplementation with a melon superoxide dismutase reduces adipose tissue in obese hamsters by improving insulin sensitivity. Mol. Nutr. Food Res..

[18] Carillon J., Romain C., Bardy G., Fouret G., Feillet-Coudray C., Gaillet S., Lacan D., Cristol J.P., Rouanet J.M. (2013). Cafeteria diet induces obesity and insulin resistance associated with oxidative stress but not with inflammation: Improvement by dietary supplementation with a melon superoxide dismutase. Free Radic. Biol. Med..

[19] Milesi M.A., Lacan D., Brosse H., Desor D., Notin C. (2009). Effect of an oral supplementation with a proprietary melon juice concentrate (Extramel) on stress and fatigue in healthy people: A pilot, double-blind, placebo-controlled clinical trial. Nutr. J..

[20] Hody S., Croisier J.-L., Bury T., Rogister B., Leprince P. (2019). Eccentric Muscle Contractions: Risks and Benefits. Front. Physiol..

[21] Nikolaidis M.G. (2017). The Effects of Resistance Exercise on Muscle Damage, Position Sense, and Blood Redox Status in Young and Elderly Individuals. Geriatrics.

[22] Proske U., Allen T.J. (2005). Damage to Skeletal Muscle from Eccentric Exercise. Exerc. Sport Sci. Rev..

[23] Proske U., Morgan D.L. (2001). Muscle damage from eccentric exercise: Mechanism, mechanical signs, adaptation and clinical applications. J. Physiol..

[24] Armstrong R.B., Ogilvie R.W., Schwane J.A. (1983). Eccentric exercise-induced injury to rat skeletal muscle. J. Appl. Physiol. Respir. Environ. Exerc. Physiol..

[25] Carillon J., Del Rio D., Teissedre P.L., Cristol J.P., Lacan D., Rouanet J.M. (2012). Antioxidant capacity and angiotensin I converting enzyme inhibitory activity of a melon concentrate rich in superoxide dismutase. Food Chem..

[26] Chiang J., Shen Y.-C., Wang Y.-H., Hou Y.-C., Chen C.-C., Liao J.-F., Yu M.-C., Juan C.-W., Liou K.-T. (2009). Honokiol protects rats against eccentric exercise-induced skeletal muscle damage by inhibiting NF-κB induced oxidative stress and inflammation. Eur. J. Pharmacol..

[27] Ruffier J.E. (1951). Considérations sur l’indice de résistance du coeur à l’effort. Med. Sport.

[28] Dickson J. (1950). L’utilisation de l’indice cardiaque de Ruffier dans le contrôle médico-sportif. Med. Sport.

[29] Cooper K.H. (1968). A means of assessing maximal oxygen intake. Correlation between field and treadmill testing. JAMA.

[30] Bolonchuk W.W. (1975). The Accuracy of the Six Minute Run Test to Measure Cardiorespiratory Fitness.

[31] Ware J.E., Sherbourne C.D. (1992). The MOS 36-item short-form health survey (SF-36). I. Conceptual framework and item selection. Med. Care.

[32] Prévost P. (2002). Le surentraînement. Sport Prépa. Phys..

[33] Tendeng M.S. (2007). Etude comparative des qualités physiques et medico-physiques d’étudiants de deuxième année après un an et trois mois de formation à l’INSEP. Mémoire de maîtrise es-sciences et technique.

[34] Kim J., Lee J. (2017). Role of transforming growth factor-beta in muscle damage and regeneration: Focused on eccentric muscle contraction. J. Exerc. Rehabil..

[35] Sato K., Li Y., Foster W., Fukushima K., Badlani N., Adachi N., Usas A., Fu F.H., Huard J. (2003). Improvement of muscle healing through enhancement of muscle regeneration and prevention of fibrosis. Muscle Nerve.

[36] Choi S.J. (2014). Cellular mechanism of eccentric-induced muscle injury and its relationship with sarcomere heterogeneity. J. Exerc. Rehabil..

[37] Saovieng S., Wu J., Huang C.Y., Kao C.L., Higgins M.F., Chuanchaiyakul R., Kuo C.H. (2018). Deep Ocean Minerals Minimize Eccentric Exercise-Induced Inflammatory Response of Rat Skeletal Muscle. Front. Physiol..

[38] Yu S.H., Huang C.Y., Lee S.D., Hsu M.F., Wang R.Y., Kao C.L., Kuo C.H. (2014). Decreased eccentric exercise-induced macrophage infiltration in skeletal muscle after supplementation with a class of ginseng-derived steroids. PLoS ONE.

[39] Zuo Q., Wang S.-C., Yu X.-K., Chao W.-W. (2018). Response of macrophages in rat skeletal muscle after eccentric exercise. Chin. J. Traumatol. Zhonghua Chuang Shang Za Zhi.

[40] Egoumenides L., Gauthier A., Barial S., Saby M., Orechenkoff C., Simoneau G., Carillon J. (2018). A Specific Melon Concentrate Exhibits Photoprotective Effects from Antioxidant Activity in Healthy Adults. Nutrients.

[41] Azad M., Khaledi N., Hedayati M. (2016). Effect of acute and chronic eccentric exercise on FOXO1 mRNA expression as fiber type transition factor in rat skeletal muscles. Gene.

[42] Liang H., Ward W.F. (2006). PGC-1alpha: A key regulator of energy metabolism. Adv. Physiol. Educ..

[43] Mortensen O.H., Frandsen L., Schjerling P., Nishimura E., Grunnet N. (2006). PGC-1alpha and PGC-1beta have both similar and distinct effects on myofiber switching toward an oxidative phenotype. Am. J. Physiol. Endocrinol. Metab..

[44] Pette D., Staron R.S. (2000). Myosin isoforms, muscle fiber types, and transitions. Microsc. Res. Tech..

[45] Puhke R., Aunola S., Ailanto P., Alev K., Venojärvi M., Rusko H., Seene T. (2006). Adaptive changes of Myosin isoforms in response to long-term strength and power training in middle-aged men. J. Sports Sci. Med..

[46] Windisch A., Gundersen K., Szabolcs M.J., Gruber H., Lømo T. (1998). Fast to slow transformation of denervated and electrically stimulated rat muscle. J. Physiol..

[47] Olesen J., Kiilerich K., Pilegaard H. (2010). PGC-1alpha-mediated adaptations in skeletal muscle. Pflug. Arch. Eur. J. Physiol..

[48] Arany Z. (2008). PGC-1 coactivators and skeletal muscle adaptations in health and disease. Curr. Opin. Genet. Dev..

[49] Cannavino J., Brocca L., Sandri M., Bottinelli R., Pellegrino M.A. (2014). PGC1-alpha over-expression prevents metabolic alterations and soleus muscle atrophy in hindlimb unloaded mice. J. Physiol..

[50] Tiraby C., Langin D. (2005). PGC-1alpha, a transcriptional coactivator involved in metabolism. Med. Sci..

[51] St-Pierre J., Drori S., Uldry M., Silvaggi J.M., Rhee J., Jäger S., Handschin C., Zheng K., Lin J., Yang W. (2006). Suppression of Reactive Oxygen Species and Neurodegeneration by the PGC-1 Transcriptional Coactivators. Cell.

[52] Carillon J., Fouret G., Feillet-Coudray C., Lacan D., Cristol J.P., Rouanet J.M. (2013). Short-term assessment of toxicological aspects, oxidative and inflammatory response to dietary melon superoxide dismutase in rats. Food Chem. Toxicol. J. Publ. Br. Ind. Biol. Res. Assoc..

[53] Rossi R., Pastorelli G., Corino C. (2013). Application of KRL test to assess total antioxidant activity in pigs: Sensitivity to dietary antioxidants. Res. Vet. Sci..

[54] Kasapis C., Thompson P.D. (2005). The Effects of Physical Activity on Serum C-Reactive Protein and Inflammatory Markers: A Systematic Review. J. Am. Coll. Cardiol..

[55] Laires M.J., Monteiro C. (2008). Exercise, magnesium and immune function. Magnes. Res..

[56] Nica A.S., Caramoci A., Vasilescu M., Ionescu A.M.D.P., Mazilu V. (2015). Magnesium supplementation in top athletes-effects and recommendations. Med. Sport. J. Rom. Sports Med. Soc..

[57] Volpe S.L. (2008). Magnesium and Athletic Performance. ACSM’s Health Fit. J..

[58] Ryan M.F. (1991). The role of magnesium in clinical biochemistry: An overview. Ann. Clin. Biochem..

[59] Nielsen F.H., Lukaski H.C. (2006). Update on the relationship between magnesium and exercise. Magnes. Res..

[60] Zhang Y., Xun P., Wang R., Mao L., He K. (2017). Can Magnesium Enhance Exercise Performance?. Nutrients.

[61] Carillon J., Notin C., Schmitt K., Simoneau G., Lacan D. (2014). Dietary supplementation with a superoxide dismutase-melon concentrate reduces stress, physical and mental fatigue in healthy people: A randomised, double-blind, placebo-controlled trial. Nutrients.

[62] Gonzalez-Gross M., Melendez A. (2013). Sedentarism, active lifestyle and sport: Impact on health and obesity prevention. Nutr. Hosp..

